# Comparative genomic analysis of seven *Mycoplasma hyosynoviae* strains

**DOI:** 10.1002/mbo3.242

**Published:** 2015-02-18

**Authors:** Eric A Bumgardner, Weerayuth Kittichotirat, Roger E Bumgarner, Paulraj K Lawrence

**Affiliations:** 1 Newport Laboratories Inc.Worthington, Minnesota; 2 Systems Biology and Bioinformatics Research Group, Pilot Plant, Development and Training Institute, King Mongkut's University of Technology ThonburiBangkhuntien, Bangkok, Thailand; 3 Department of Microbiology, University of WashingtonSeattle, Washington

**Keywords:** Adhesin, antibiotic, CRISPR, genome, MAA1, *Mycoplasma hyosynoviae*, OppA, phage, resistance

## Abstract

Infection with *Mycoplasma hyosynoviae* can result in debilitating arthritis in pigs, particularly those aged 10 weeks or older. Strategies for controlling this pathogen are becoming increasingly important due to the rise in the number of cases of arthritis that have been attributed to infection in recent years. In order to begin to develop interventions to prevent arthritis caused by *M. hyosynoviae*, more information regarding the specific proteins and potential virulence factors that its genome encodes was needed. However, the genome of this emerging swine pathogen had not been sequenced previously. In this report, we present a comparative analysis of the genomes of seven strains of *M. hyosynoviae* isolated from different locations in North America during the years 2010 to 2013. We identified several putative virulence factors that may contribute to the ability of this pathogen to adhere to host cells. Additionally, we discovered several prophage genes present within the genomes of three strains that show significant similarity to MAV1, a phage isolated from the related species, *M. arthritidis*. We also identified CRISPR-Cas and type III restriction and modification systems present in two strains that may contribute to their ability to defend against phage infection.

## Introduction

*Mycoplasma hyosynoviae* is an economically important pathogen of swine that causes nonsuppurative arthritis, most typically in pigs aged 10 weeks or older (Hagedorn-Olsen et al. 1999a,b). Like most *Mollicutes* sp. *M. hyosynoviae* lacks a cell wall, and is dependent on its host for numerous nutritional requirements (Razin and Freundt 1984). *Mycoplasma hyosynoviae* is distributed worldwide and has been isolated in many parts of Europe (Blowey 1993; Kokotovic et al. 2002), North America (Blowey 1993), Asia (Kokotovic et al. 2002), and Australia (Furlong and Turner 1975). Recent evidence indicates that the prevalence of arthritis related to infection with *M. hyosynoviae* has been increasing in the United States over the course of the past decade (Gomes Neto et al. 2012).

Pigs commonly become infected with *M. hyosynoviae* through exposure to nasal excretions from chronically infected animals. Once an animal is exposed, the organism will rapidly colonize the tonsils, from whence it is commonly isolated in adult pigs. *Mycoplasma hyosynoviae* has been shown to spread systemically through the blood stream to the joints of infected animals during acute infection (Hagedorn-Olsen et al. 1999a,b). During systemic spread cells may attach to the synovial membrane within the joint and ultimately cause inflammation and arthritic symptoms. The molecular basis for attachment to the synovial membrane remains largely unknown. However, previous studies involving the related organism *M. arthritidis* implicated a 90 kDa adhesin, MAA1, in attachment of bacterial cells to the surface of rat synovial fibroblasts (Washburn et al. 1993, 2000).

Phylogenetic analyses performed thus far using an amplified fragment length polymorphism technique have revealed a great breadth of diversity among isolates (Kokotovic et al. 2002). Even when strains are isolated from the same site, a high degree of genomic variability has been shown to occur (Kokotovic et al. 2002). In the same study, genomic characterization based on sequencing of amplified 16S rRNA sequences demonstrated limited variability between different *M. hyosynoviae* strains. In order to more fully characterize the genetic composition, strain diversity, nutritional requirements, and putative virulence factors of this emerging pathogen, a study to elucidate the full genome sequence of recent strains was needed.

The present study reports, for the first time, the draft genome sequences of seven *M. hyosynoviae* strains isolated at different dates and from different regions within North America. In comparing and contrasting these sequences, we have identified several genes that appear to be unique to particular strains including a clustered regularly interspaced short palindromic repeats, and multifunctional protein complex (Cas proteins), CRISPR-Cas system, and a type III restriction and modification system as well as a number of prophage sequences. In addition, we identified several genes that appear to bear homology to known virulence factors such as the adhesins MAA1 and OppA, as well as several genes involved in resistance to fluoroquinolone antibiotics in other *Mycoplasma* species.

## Materials and Methods

### Isolation of *M. hyosynoviae* strains

*Mycoplasma hyosynoviae* isolates were obtained from samples taken from pigs at several locations and dates as described in Table1. All isolates were obtained from the joints of pigs exhibiting symptoms of arthritis. Samples were first diluted in a small volume of Dulbecco's Modified Eagle's Medium (DMEM) and passed through a 0.22 *μ*m filter to remove larger bacteria present in the sample. A portion of the filtered sample was then used for polymerase chain reaction (PCR) to verify the presence of the organism. One milliliter of filtered fluids was inoculated into 9 mL of Mycoplasma medium (Pleuropneumonia Like Organism (PPLO) broth with 5% w/v mucin and 20% v/v porcine serum) and incubated at 37°C for 3–7 days. After the initial culture 1 mL of the fluids was plated on blood agar to monitor for purity and a second sample of culture fluid was tested for the presence of *M. hyosynoviae* by qPCR (quantitative PCR). Next, the culture and sampling procedures were repeated for an additional passage and aliquots of the culture were frozen after addition of glycerol for preservation. These aliquots were stored at −70°C until they were removed to grow cultures of each strain for isolation of genomic DNA.

**Table 1:** Description of *Mycoplasma hyosynoviae* strains

Strain	Location isolated	Date	Tissue
NPL1	Fargo, ND	15 October 2010	Joint fluid
NPL2	Cambridge, Ontario, Canada	25 June 2013	Joint fluid
NPL3	Mexico, IN	25 October 2012	Joint swab
NPL4	Kalona, IA	25 August 2010	Joint fluid
NPL5	Britt, IA	20 September 2012	Joint fluid
NPL6	Taylor, AZ	16 February 2011	Joint swab pool
NPL7	Worthington, MN	29 May 2013	Joint swab

### qPCR to verify the presence of *M. hyosynoviae*

qPCR was used to verify the presence of *M. hyosynoviae* isolates and also to monitor the growth of the organism in culture. Samples were processed for use in qPCR by centrifuging 1 mL of culture for 5 min at 10,000*g*. The supernatant was removed and discarded. The pellet was resuspended in 100 *μ*L of Prepman® (Life Technologies, Carlsbad, CA, USA) reagent and boiled for 10 min. The boiled preparation was then centrifuged for 5 min at maximum speed and 50 *μ*L of supernatant was removed for use in qPCR. Samples were resuspended in master mix containing primers and probes at 5 and 2.5 nmol/L concentrations, respectively, using 2X buffer (Qiagen Quantitect®, Qiagen, Valencia, CA, USA), molecular grade water, and 2.5 *μ*L of the extracted sample.

The primer and probe set used was as follows:

Probe: /5HEX/TGTGCAGTGTCACGGTTAAGACCA/3IABlkFQ/
Forward primer: ATAACTTCGCTTGGACCTCG
Reverse primer: GTCCAATGTTAGGTCCTTCAGG

PCR was performed under the following conditions:

95°C/15 min
45 cycles: 94°C/15 sec
60°C/60 sec.

Results were reported as C*t* values. Any sample with a C*t* less than 37 was considered positive.

### Preparation of genomic DNA and sequencing

*Mycoplasma hyosynoviae* strains were inoculated into mycoplasma culture medium and grown for 3 days at 37°C. A 10 mL volume of each culture was then centrifuged at 7500*g* for 20 min in order to pellet cells. Following this centrifugation the supernatant was discarded and genomic DNA was obtained from the cell pellet by phenol:chloroform extraction and ethanol precipitation (Green and Sambrook 2012a,b). Preparation of sequencing libraries and sequencing of genomic DNA was performed at the University of Washington on an Illumina MiSeq® (Illumina, San Diego, CA, USA) instrument as per the manufacturer's instructions for a paired end 250 bp library.

### De novo sequence assembly, gene prediction, and annotation

FastQ files containing forward and reverse reads obtained from each strain were uploaded into the Orione®instance of Galaxy (Cuccuru et al. 2014). De Novo assembly of contiguous sequences was then performed using Velvet Optimizer (Zerbino and Birney 2008) and SPAdes (Bankevich et al. 2012). Contig files from Velvet® and scaffold files from SPAdes were then uploaded into CISA (Lin and Liao 2013) for each strain. The output from CISA consisted of integrated supercontigs and was downloaded from Galaxy for use in further analysis. The supercontigs from each strain were aligned with the genome of *M. arthritidis* strain 158L3-1 (NCBI: CP001047) using Mauve contig mover (Rissman et al. 2009). *Mycoplasma arthritidis* was used because multiple BLAST searches using the largest contigs from each assembly consistently returned *M. arthritidis* as the closest match. The ordered multi-FASTA file obtained from this program was uploaded into Artemis (Rutherford et al. 2000). The final draft genome sequence was then written out in FASTA format. The genomes from each strain were uploaded onto the RAST website (Aziz et al. 2008) for gene prediction and annotation. Annotated versions of each strain were then downloaded and used in further analysis.

### Comparison of annotated genomes

**Figure 1:**
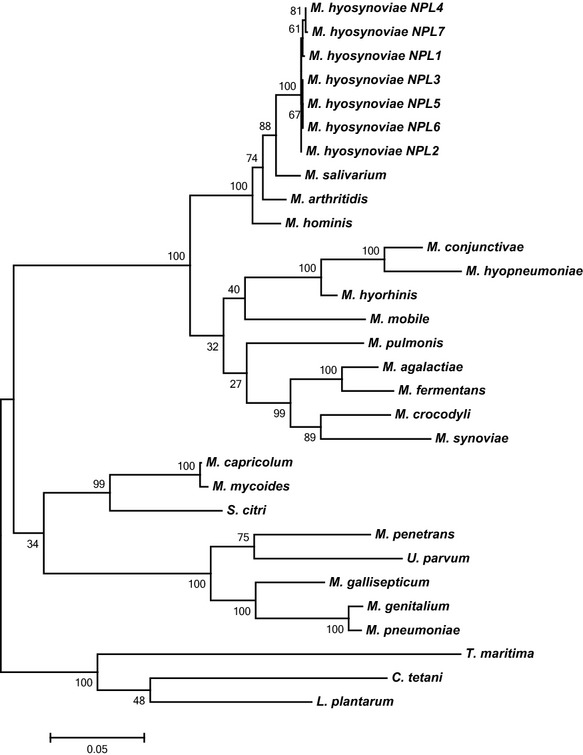
Comparison of 16S rRNA sequences in Mycoplasmatacea. Maximum likelihood tree of 16S rRNA sequences. Sequences were obtained from the NCBI database with the following accession numbers: *Mycoplasma hyosynoviae* NPL1 (NCBI: JFKL01000049.1), NPL2 (NCBI: JFKK01000042.1), NPL3 (NCBI: JFKJ01000029.1), NPL4 (NCBI: JFKI01000009.1), NPL5 (NCBI: JFKM01000003.1), NPL6 (NCBI: JFKH01000034.1), NPL7 (NCBI: JFKG01000007.1), *M. salivarium* (NCBI: NZ_AXZE01000009.1), *M. arthritidis* (NCBI: NR_102867.1), *M. hominis* (NCBI: M96660.1), *M. conjunctivae* (NCBI: NR_074135.1), *M. hyopneumoniae* (NCBI: Y00149.1), *M. hyorhinis* (NCBI: CP002170.1), *M. mobile* (NCBI: NR_074620.1), *M. pulmonis* (NCBI: NC_002771.1), *M. agalactiae* (NCBI: AJ419900.1), *M. fermentans* (NCBI: NR_102937.1), *M. crocodyli* (NCBI: NR_074301.1), *M. synoviae* (NCBI: NR_044811.1), *M. capricolum* (NCBI: U26048.1), *M. mycoides* (NCBI: NR_074703.1), *Spiroplasma citri* (NCBI: NR_036849.1), *M. penetrans* (NCBI: NR_074145.1), *Ureaplasma parvum* (NCBI: NR_074762.1), *M. gallisepticum* (NCBI: NR_074433.1), *M. genitalium* (NCBI: NR_074611.1), *M. pneumoniae* (NCBI: NR_074554.1), *Thermotoga maritima* (NCBI: NR_102775.1), *Clostridium tetani* (NCBI: X74770.1), *Lactobacillus plantarum* (NCBI: FR745400.1).

16S rRNA sequences from several strains of related bacteria were downloaded from GenBank® and aligned with all seven strains of *M. hyosynoviae* using ClustalW2 (Larkin et al. 2007) on the Orione® instance of Galaxy (Cuccuru et al. 2014). A phylogenetic analysis was conducted using a maximum likelihood tree in MEGA® version 6 with 1000 bootstrap replicates (Tamura et al. 2013) (Fig.1). Two way comparisons were performed between each of the genomes from the seven strains of *M. hyosynoviae* described here using the functional gene comparison utility on RAST's SEED® server. Differences between strains were manually condensed. Metabolic pathway comparisons were performed using the SEED website based on KEGG® pathways (Overbeek et al. 2005; Kanehisa and Goto 2000) and subsystems. A phage sequence detection tool known as PHAST was used to search each annotated genome for both complete and incomplete prophage sequences (Zhou et al. 2011). CRISPR sequences were detected using CRISPRfinder (Grissa et al. 2007). Contigs derived from all seven strains were aligned for comparison (Data S1) using Mauve.

### Comparison of Mollicutes CRISPR loci

In order to compare the CRISPR loci of different Mollicutes species, we first searched the NCBI database using the search term “CRISPR associated” and then restricted the search to Mollicutes. This search identified sequences in several species including *M. arginini*, *M. iowae*, *M. imitans*, *M. spumans*, *M. salivarium*, *Spiroplasma syrphidicola*, *Spiroplasma apis*, *Candidatus hepatoplasma crinochetorum*, *M. ovipneumoniae*, *M. synoviae*, *M. arthritidis*, *M. mobile*, *M. canis*, *M. cynos*, *M. lipofaciens*, *Acholeplasma palmae*, *M. hyosynoviae*, and several strains of *M. gallisepticum*. However, several of these species contained truncations in their CRISPR loci due to gaps in the assembly. In many cases, *Cas* genes could be identified but no CRISPR array was detectable using CRISPRfinder. Therefore, we restricted our analysis to only those species in which we could identify *Cas* genes as well as an identifiable CRISPR array. This restricted our analysis to sequences derived from *A. palmae* J233 (NCBI: FO681347.1), *C. hepatoplasma crinochetorum* Av (NCBI: CP006932.1), *M. canis* PG14 (NCBI: NZ_AJFQ01000005.1), *M. ovipneumoniae* SC01 (NCBI: NZ_AFHO01000003.1), *M. synoviae* 53 (NCBI: NC_007294.1), *M. arthritidis* 158L3-1 (NCBI: CP001047.1), *M. cynos* C142 (NCBI: NC_019949.1), *M. gallisepticum* NY01 (NCBI: NC_018409.1), *M. mobile* 163K (NCBI: NC_006908.1), *M. hyosynoviae* NPL1 (NCBI: JFKL01000035.1), and *S. apis* B31 (NCBI: CP006682.1). In addition, we restricted our analysis to only one strain of each species. In many cases, CRISPR loci were not annotated in the database so CRISPRfinder was used to identify the CRISPR arrays present in each strain. HMMER (Finn et al. 2011) was used to identify *Cas* gene homologs missing from the original annotation by manually searching genes surrounding CRISPR arrays. The resulting loci were downloaded from the NCBI database and were compared and visualized using Easy Fig 2.1 (Sullivan et al. 2011). An *E*-value of 10^−9^, and a minimum sequence length of 40 bp were used in the comparison (Fig. 4).

## Results and Discussion

### Sequence assembly and comparison

The metrics obtained during assembly of the seven strains of *M. hyosynoviae* described in this study are described in Table2. We were able to obtain a high level of coverage for these genomes at a minimum of 38× and a maximum of 133× between strains. During the assembly it was apparent that the scaffolds generated using SPAdes® were generally longer than those obtained using Velvet® Optimizer and consequently fewer contigs were obtained using this method. The N50 values also are generally higher when using SPAdes; however, during assembly of two of the strains, Velvet was able to obtain higher N50 values. Nonetheless, integration of contigs from both assemblers using CISA generally appeared to favor the SPAdes output in terms of the length of the longest contig and overall distribution in contig size as measured by N50. An alignment of the contigs from each strain (Fig. S1) revealed several potential genome rearrangements. In particular, the largest contig from each strain consists of two to three large locally collinear blocks (LCBs) assigned by Mauve. All three blocks are part of a ∽480 kB contig in the NPL1 strain and remain together in all strains with the exception of NPL6. In this strain, two contigs are aligned between one of the LCBs and the remaining two. It is possible that this putative rearrangement may affect gene regulation and expression in the NPL6 strain. However, the edges of the LCBs involved in this rearrangement are within regions of the genome that encode hypothetical proteins, so it is difficult to know what affect such a rearrangement might have.

**Table 2:** Assembly metrics of MiSeq data for each *Mycoplasma hyosynoviae* strain

Strain	Paired reads	Estimated coverage	Final contig size range (kb)	# velvet contigs	Velvet N50	# spades scaffolds	Spades N50	CISA # contigs	CISA N50
NPL1	443,590	129x	44.7–481.7	33	208,446	25	120,343	6	481,749
NPL2	304,852	81x	7.6–366.7	45	128,654	39	129,749	10	129,749
NPL3	462,448	133x	1.3–357.4	20	155,064	14	195,536	9	195,535
NPL4	136,406	38x	23.4–357.2	19	134,693	17	168,980	8	168,980
NPL5	200,300	60x	65.6–264.5	211	98,397	23	195,537	7	195,537
NPL6	367,168	103x	15.7–280.2	21	210,057	22	195,533	8	194,315
NPL7	214,296	58x	4.8–360.2	23	151,365	28	175,555	8	177,387

Coverage was estimated based on a read length of 250 bp, multiplied by the number of reads, divided by the size of the resulting genome. All other metrics were derived from the output of the specific program described.

### Comparison of *M. hyosynoviae* genomes

The size of the *M. hyosynoviae* genomes sequenced in this study ranged from 858,952 bp for strain NPL1 to 936,147 bp for strain NPL2. This is a typical size range for a *Mycoplasma* species; however, the large variability in the size of each genome was surprising. Several phage insertions were noted, particularly in the genome of strain NPL4 and these contribute to the differences in the size of the genome. In terms of the degree of relatedness to other species of *Mycoplasma* it appears that *M. salivarium* is the closest relative of *M. hyosynoviae* available in the database at the present time as determined by comparing the sequences for 16S ribosomal subunits (Fig.1). Nonetheless, the distance between these strains and those of other *Mycoplasma* species may indicate that several intermediate species could be present in the environment. As depicted in Table3, GC content was typical for *Mycoplasma* species, at about 27% for each strain sequenced. Average gene length remained consistent between strains at ∽1150 bp. The gene density of each genome remained largely constant at about 90% within each genome. The number of coding sequences present in each genome ranged from 707 to 765. Many of the additional proteins appear to be annotated as hypothetical as few differences were noted in the functionally annotated genes present (Table4). Locus number information for all genes that are specifically discussed in the Results and Discussion section is included in Table S1. Only two rRNAs were identified in each genome consisting of 23S and 16S subunits. In contrast, the number of tRNAs present in each strain varied from 25 in strain NPL5 to 41 in NPL6 which encodes six copies of methionine tRNA. When comparing annotated genes among *M. hyosynoviae* genomes, the vast majority of genes were present in all seven strains. However, as shown in Table4, some differences were noted. These include the presence of CRISPR-associated proteins and the type III restriction and modification system that was peculiar to strains NPL1 and NPL2. The remaining proteins, which were only absent in one strain, may have been missed during the assembly or may represent gene deletion events. The presence of the CRISPR-associated proteins and type III restriction and modification system within the genomes of strains NPL1 and NPL2 is likely used to help prevent infection with bacteriophage.

**Table 3:** Genome characteristics of individual *Mycoplasma hyosynoviae* strains

Isolate	Genome size	G + C content (%)	# features (genes)	Coding sequences	Gene density (%)	Average gene length (bp)	CDS with predicted function	#tRNAs	#rRNAs
NPL1	858,952	26.8	707	672	90.5	1156	267	33	2
NPL2	936,147	27.4	752	715	89.7	1174	318	35	2
NPL3	868,007	27.1	714	679	90.9	1162	298	33	2
NPL4	885,162	27	724	689	90.9	1168	296	33	2
NPL5	918,172	27.1	744	717	90.3	1156	298	25	2
NPL6	886,543	27.1	720	677	89	1166	323	41	2
NPL7	927,803	27.1	765	724	90.8	1163	352	39	2

**Table 4:** Absence or presence of genes across *Mycoplasma hyosynoviae* strains

Gene	NPL1	NPL2	NPL3	NPL4	NPL5	NPL6	NPL7
YebC	−	+	+	+	+	+	+
cas1	+	+	−	−	−	−	−
cas2	+	+	−	−	−	−	−
csn1	+	+	−	−	−	−	−
DNA-cytosine methyltransferase	+	−	+	+	+	+	+
Type III restriction DNA endonuclease	+	+	−	−	−	−	−
Type III restriction methylation subunit	+	+	−	−	−	−	−
S14p	−	+	+	+	+	+	+
S14p, Zn dependent	−	+	+	+	+	+	+

“+” indicates that a gene was present in a strain; “−” indicates that the gene was absent in a strain.

### Prophage strains

**Figure 2:**
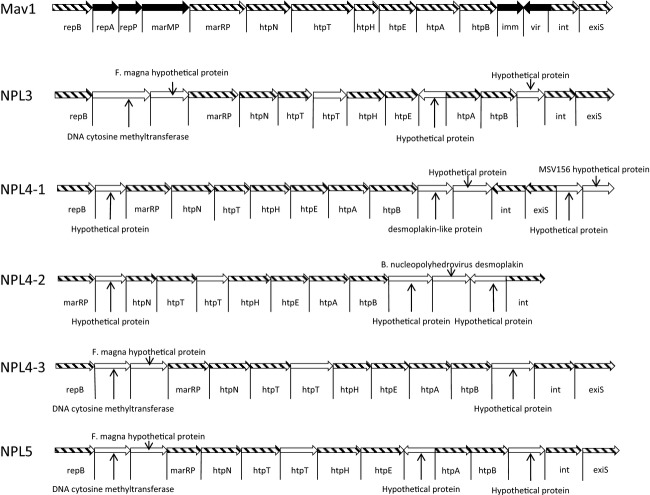
Structure of prophage genomes identified in *Mycoplasma hyosynoviae* strains. Comparison of prophage genomes identified in *M. hyosynoviae* strains. MAV1 phage is included for comparison. Genes depicted in black are those not shared by *M. hyosynoviae*-derived phage. Genes highlighted in white are unique to *M. hyosynoviae*-derived phage strains and are not present in MAV1. Genes displayed with a cross-hatch pattern are those shared between *M. hyosynoviae*-derived prophage and MAV1 phage.

*Mycoplasma hyosynoviae* strains NPL3, NPL4, and NPL5 all displayed at least one phage insertion within the genome. In all cases the closest match to the identified phage strain was to MAV1 (NCBI: NC001942) or PhiMFV1 (NCBI: NC005964) mycoplasma phage strains which both encode 10 proteins with significant similarity to the identified sequence in all of the prophage identified. MAV1 is a strain of phage known to infect *M. arthritidis*, a close relative of *M. hyosynoviae* in the Mollicutes class, while phiMFV1 is a strain known to infect *M. fermentans*, another close relative of *M. hyosynoviae*. Strain NPL4 was the most heavily infected strain. This strain possessed two complete prophage sequences as well as another high confidence match to an incomplete version of a phage genome. Strains NPL3 and NPL5 both possess a single identical 15.9 kb prophage. The organization of the genes within the strain NPL3 and NPL5 prophage bears a strong resemblance to that of the MAV1 phage as depicted in Figure2. However, in the prophage identified here, no Vir, Imm, RepA, and RepP homologs could be found. The vir protein has been shown to encode resistance to further infection by MAV1 phage and has been implicated as a virulence factor during infection of rats with *M. arthritidis* (Roske et al. 2004). Imm is a repressor protein that maintains lysogeny during infection. The functional roles of the *repA* and *repP* genes are currently unknown. Additionally, in the *M. hyosynoviae* prophage, a DNA-(cytosine-5)-methyltransferase analogous to the MarMP protein in MAV1 is encoded immediately following the RepB protein. Immediately following the DNA-(cytosine5)-methyltransferase is an open reading frame (ORF) that encodes a protein with homology to a hypothetical protein encoded in a plasmid isolated from *Finegoldia magna* (NCBI: YP001691054). *Finegoldia magna* is a commensal organism that is known to cause arthritis in humans (Levy et al. 2009). In addition to these, the prophage sequence appears to contain an extra copy of a protein with some homology to HtpT as well as two other hypothetical proteins with no homology to anything currently in the database. Two of the phage sequences identified in strain NPL4 differ significantly from those identified in strains NPL3 and NPL5 based on homology at the nucleotide level, while one of the phage is identical except that it is missing a hypothetical protein that is inserted between htpE and htpA in the phage from the NPL3 and NPL5 strains. Nonetheless, all of the phage described in this report have a similar genomic structure as can be seen in Figure2. Mainly, differences in genomic structure for the phage identified in strain NPL4 are in the number and type of hypothetical proteins present. Interestingly, the phage insertions in strains three and five as well as the NPL 4-3 phage shown in Figure2 correspond to the location of truncated CRISPR sequences identified in these strains. These sequences map to the central portion of the *F. magna* hypothetical protein within the prophage genome. Thus, it seems possible that these repeats have been targeted by the phage as an integration site. Indeed, the direct repeat sequence contains a TTTTA sequence similar to that used by MAV1 phage for insertion into the genome of *M. arthritidis* (Roske et al. 2004).

### DNA replication and repair

In addition to possessing DNA polymerases I, III, and IV, every strain of *M. hyosynoviae* sequenced during the course of this study possesses a set of genes encoding subunits A and B of topoisomerase IV. In each genome, these genes are adjacent to one another. The NPL2 strain possesses two copies of each of these genes which are located together in two different regions of the genome. Each strain also possesses a copy of topoisomerase I. These genes are responsible for unraveling supercoiled DNA and separating daughter chromosomes during replication.

Each strain also encodes a large number of enzymes associated with DNA repair. All seven strains possess copies of RecA, DinP, Ssb, EndoIV, and Ogt enzymes. RecA is a canonical enzyme induced in the SOS response to stress in many prokaryotes and also functions in DNA repair. In particular, *M. hyosynoviae* appear to be sensitive to heat shock and encodes a large complement of genes to mediate against this particular stressor. DinP, or DNA polymerase IV, is an error-prone polymerase with no 3′ to 5′ exonuclease activity that is commonly induced in response to DNA damage within the cell. Ssb is a single-stranded DNA-binding protein with multiple known functions related to the cold-shock response, DNA replication, and DNA repair in *Escherichia coli* (Meyer and Laine 1990) and a homolog of this protein has been identified in *M. pneumoniae* (Das et al. 2007). During DNA repair, this protein is expected to be responsible for the binding of single-stranded DNA after damage has occurred to prevent its subsequent degradation by the cell and enable genome repair. Structural predictions have indicated that it may possess RNA binding activity and it may have the ability to bind tRNA (Das et al. 2007). EndoIV is an endonuclease and is also known in *Mycoplasma* species as Nfo. It has been shown to be encoded in *M. gallisepticum* (Gorbachev et al. 2013). This protein functions as an endonuclease that targets oxidized nucleotides within the DNA strand and makes a single-stranded excision which is then repaired by DNA polymerase. The *ogt* gene codes for the protein, 6-*O*-methyl-guanine-transferase which has been identified in at least three other *Mycoplasma* species (Carvalho et al. 2005). This protein is used to repair methylation of DNA by alkylating agents such as Mitomycin C which is often used to prevent *Mycoplasma* infections in cell culture. Strains NPL1, NPL3, NPL4, NPL5, NPL6, and NPL7 possess a copy of a cytosine methyltransferase and all strains encode a copy of a gene labeled as an adenosine methyltransferase. These proteins have been shown to function as a means of regulating chromosomal replication in *E. coli* (Lluch-Senar et al. 2013).

All seven strains of *M. hyosynoviae* presented here encode copies of formamidopyrimidine-DNA glycosylase, uracil-DNA glycosylase, and DNA3-methyladenine glycosylase which are all involved in excising damaged bases from the nuclear backbone. Previous studies in *M. gallisepticum*, *M. capricolum*, and in *M. lactucae* have noted that uracil-DNA glycosylase is not as efficient in *Mycoplasma* species (Williams and Pollack 1990). It has been suggested that this is the cause of the high degree of AT bias in most species of *Mycoplasma*. *Mycoplasma hyosynoviae* is no exception, having a GC content of approximately 27%. Further research into the kinetics of the enzyme encoded by *M. hyosynoviae* should be performed to determine whether the uracil-DNA glycosylase encoded by this species excises damaged bases inefficiently as well.

In all seven strains, the DNA polymerase I gene is encoded immediately adjacent to the DNA polymerase III *α* subunit. This gene and the architecture of its operon bear a strong resemblance to that of other *Mycoplasma* species such as *M. hyopneumoniae* and *M. arthritidis*.

One common mechanism for DNA repair in most prokaryotes is the RecFOR or RecOR system that repairs single-stranded breaks in the genome. *Mycoplasma hyosynoviae* appears to mediate repair through the use of a complete RecOR recombination system as all seven strains encode RecA, RecO, RecR, and Ssb but lack RecF. All seven strains also possess YrrC, a RecD-like DNA helicase homolog. Whether this enzyme functions as an active helicase in *Mycoplasma* has never been established and its relevance to DNA repair and recombination in the RecOR system remains unknown. Additionally, all seven strains encode a copy of hyp2 which is a hypothetical protein encoded between the gamma and tau subunits of DNA polymerase III and RecR; however, the role of this protein in DNA repair is also unknown.

All of the strains analyzed here possess an uvrABCD DNA repair system. The *uvr* genes are a part of an SOS system that responds to DNA damage that has been described for *M. gallisepticum* previously (Gorbachev et al. 2013). Each protein performs a different function with regard to DNA repair. UvrA binds to damaged DNA, UvrB pulls the strands apart, UvrC excises the damaged nucleotides, and UvrD binds to the excised nucleotides and carries them away. This system is a common means of DNA repair in prokaryotes and its presence in *M. hyosynoviae* is not surprising. Without an *uvr* operon mutations would be much more frequent and would likely eventually result in an evolutionary dead end for any strain lacking this means of repair.

All of the strains sequenced during the course of this study possess a complete type I restriction modification system encoding hsd subunits M, R, and S. The system is used as a means of defense against phage infection and the three different subunits are responsible for different components of target recognition and destruction of nonmethylated restriction sequences. The S subunit is involved in target recognition and binds to target DNA. The M subunit is responsible for methylation of target sequences which serves to silence phage genes, and the R subunit is responsible for endonucleolysis effectively removing target sequences to which the complex binds. This system has been previously identified in *M. pulmonis* (Sitaraman and Dybvig 1997), however in *M. hyosynoviae* it is restricted to a single locus and only one copy of subunit S is encoded.

Strains NPL1 and NPL2 uniquely encode a type III restriction and modification system similar to one reported for *M. pulmonis* (Dybvig et al. 2007). Much like the system reported for *M. pulmonis*, the system in *M. hyosynoviae* consists of two modification subunits encoded immediately prior to the restriction subunit. However, in *M. hyosynoviae* the first modification subunit is severely truncated consisting of only 104 amino acids, whereas the first modification subunit in the *M. pulmonis* genome encodes a protein 653 amino acids in length.

All seven strains possess genes encoding two DNA structural proteins known as HimAB (an integration host factor) and Smc (chromosome partition protein). HimAB was originally identified as a host susceptibility factor for phage infection in *E. coli*, but has since been assigned a more structural role. Proteins in this family are known to condense nuclear DNA performing a role similar to that of histones in eukaryotic cells. Smc protein has been identified as playing a critical role in the separation of bacterial chromosomes during replication of *Bacillus subtilis* (Britton et al. 1998) and is thought to be involved in nucleoid condensation. Homologs of this protein have been identified in other species of *Mycoplasma* including *M. arthritidis*, *M. genitalium*, *M. pneumoniae*, and *M. hyorhinis*. The region of the genome in which the gene is mapped for *M. hyosynoviae* bears a strong similarity to that of the *M. arthritidis* genome.

### RNA metabolism

As described for *M. hyopneumoniae* and *M. synoviae* (Madeira and Gabriel 2007), *M. hyosynoviae* possesses an RNA transcription system consisting of the alpha, beta, beta’, and RpoD RNA polymerase subunits as well as NusA, and GreA, transcription elongation factors. In addition an antitermination factor, NusG, is also present in all seven of the *M. hyosynoviae* genomes that we sequenced. However, all seven strains also possess a NusB transcription termination protein very similar in location and size to a hypothetical protein expressed by *M. arthritidis*. This feature is not found in *M. hyopneumoniae* or *M. synoviae*. Furthermore, all seven strains possess a small protein with a predicted nucleic acid binding function labeled as COG2740 encoded immediately upstream of NusA. Also of interest is the presence of a transcription accessory protein with an S1-RNA binding domain immediately downstream of NusA. This feature can also be found in the genomes of *M. synoviae*, *M. arthritidis*, and *M. agalactiae*; however, the location of the gene is different in *M. synoviae* and *M. agalactiae*. In the NPL4 strain, a hypothetical protein similar in size to the NusA protein is located in the same region as *nusA* for the remaining strains in the study; however, RAST was unable to assign a name to this protein as it appears to share slightly less homology to *nusA* encoded by other strains of Mycoplasma.

*Mycoplasma hyosynoviae* appears to encode seven different types of RNAse including an RNAseIII, RNAseR, and bacillus type ribonucleases, NrnA, J1, J2, and RnhC. The RNAse R gene encodes an exoribonuclease that is thought to perform numerous functions in RNA metabolism in *M. genitalium* (Lalonde et al. 2007). Two *nrnA* type ribonucleases are encoded sequentially in all seven strains presented here with a third *nrnA* ribonuclease further upstream. Interestingly, NPL2 contains two separate identical segments in which the triplicate set of *nrnA* type ribonucleases is encoded. The function of the NrnA protein has been characterized in *M. pneumonia*e as having a bifunctional role in degradation of nanoRNA and dephosphorylation of 3′-phosphoadenosine 5′-phosphate to AMP (Postic et al. 2012). A *nrnA* gene can also be found in *M. arthritidis*, *M. pulmonis*, and *M. hyopneumoniae*. However, only *M. hyosynoviae* encodes a minimum of three *nrnA* type ribonucleases, suggesting that NrnA plays a critical role in RNA metabolism for the cell. The presence of *rnhC* in the genome of *M. hyosynoviae* is an interesting finding. The roles of this gene and its protein are not well described within *Mycoplasma* species; however, in *B. subtilis* the RnhC protein is involved in degradation of RNA–DNA hybrids (Ohtani et al. 1999). RNAses J1 and J2 are thought to play homologous roles in *B. subtilis* to RNAses E and G in *E. coli* (Kaberdin et al. 2011). Both of these genes can also be found in the genomes of *M. arthritidis* and *M. pulmonis* as well suggesting that *Mycoplasma* species may encode a large subset of ribonucleases.

All strains of *M. hyosynoviae* sequenced in this study possess genes for *mraZ*, *rsmH*, and *ftsZ* encoded next to one another with a conserved hypothetical protein encoded between *rsmH* and *ftsZ*. The role of the hypothetical protein remains unknown, however, it is likely part of the same operon and transcription of this gene would be regulated similarly to that of the other functionally annotated genes which play important roles in cell division (Alarcon et al. 2007). In addition, to their function in cell division MraZ and RsmH also function as rRNA methyltransferases that modify sites within the P site of the ribosome. Another rRNA methyltransferase with similar activity, RsmI, is also present within the genome and is encoded upstream of the remaining components of this system.

All seven NPL strains analyzed in this study encode a cysteine desulfurase, a *mnmE* gene, and a *gidA* gene. The proteins MnmE and GidA function as enzymes to add carboxymethylaminomethyl groups to U34. This modification is required for ribosomes to read codons containing wobble base pairs in RNA molecules (De Crecy-Lagard et al. 2012) and may be critical in *Mycoplasma* species due to the limited number of tRNAs encoded in their genomes. The cysteine desulfurase encoded by these strains is likely involved mainly in iron metabolism as the gene appears to be encoded within a locus dedicated to iron and sulfur metabolism. This finding is consistent with the organization of this portion of the genome within *M. agalactiae* (Baranowski et al. 2010) and similar genes are also encoded at the same locus in *M. arthritidis*. However, cysteine desulfurases can also function to modify tRNAs (Mihara and Esaki 2002).

Interestingly, all seven strains encode a unique cca tRNA nucleotidyltransferase which has not been reported previously in *Mycoplasma* species. A PSI-BLAST search was performed using the protein sequence derived from this gene and the results revealed the presence of a nudix hydrolase motif in the N-terminal portion of the protein which is consistent with a bifunctional role in cca addition. One domain of the enzyme could function in hydrolysis of nucleotides from host substrates while another could serve to add cca sequences to the 3′ end of tRNAs, possibly increasing translation efficiency.

### Potassium metabolism

All seven strains possess a copy of *ktrA* and *ktrB* genes which encode potassium uptake proteins. These genes are located adjacent to one another within each of the genomes and are likely transcribed from the same operon. Both genes have been previously identified in the genomes of *M. pneumoniae* and *M. genitalium* (Nakamura et al. 1998). In these as well as other bacterial species, these proteins play an important role in the maintenance of osmotic pressure and pH within the cell as they are all capable of high-affinity binding to potassium and are very efficient at transporting it across the cell membrane. In addition, all seven strains also encode a gene for a large-conductance mechanosensitive channel (mscL). This protein has been shown to function as an ion pore that is activated in response to mechanical stress (Oakley et al. 1999). As cells experience stress, particularly in response to stretching of the membrane, the channel is opened to allow the exchange of ions through the channel and relieve osmotic pressure, preserving cellular integrity.

### Nutrient transport

**Figure 3:**
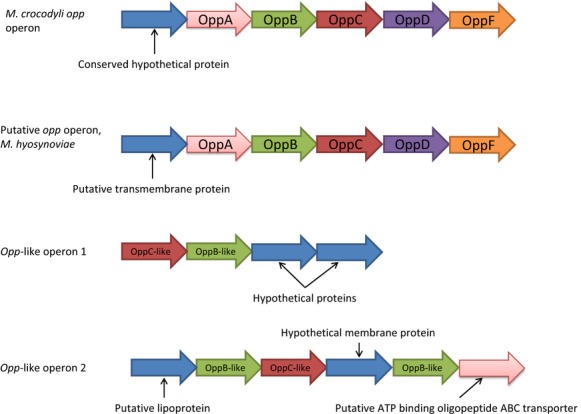
Structure of putative *opp* operon and *opp*-like operons identified in *Mycoplasma hyosynoviae* in comparison to the *opp* operon of *M. crocodyli*.

Most *Mycoplasma* species lack the enzymes necessary to perform many of the fundamental reactions that produce amino acids and degrade sugars as well as other substrates. These are necessary functions for any living cell and *Mycoplasma* species depend on their host to supply these nutrients to them. *Mycoplasma hyosynoviae* is no exception and encodes several genes dedicated to the procurement of biologically available nutrients from its host. Encoded in all seven of the strains, we sequenced in this study is an *opp* operon consisting of genes *oppA*, *oppB*, *oppC*, *oppD*, and *oppF*. All seven strains encode two additional operons containing proteins annotated as OppC and OppB by RAST and these are surrounded by hypothetical membrane proteins. The translated sequences from these genes are markedly different than that of the OppC and OppB proteins associated with the classical *opp* operon. Based on the annotation of the proteins present and the similarity of these operons to that of the *opp* operons in *M. crocodyli* (NCBI: CP001991.1) (Fig.3) and other *Mycoplasma* species, these operons likely function as ABC transporters as well. The *opp* operon has been characterized as a family of proteins that function to bring oligopeptides and ATP across the cell membrane for use as nutrients and building blocks in the cell (Henrich et al. 1999; Oakley et al. 1999). The presence of two putative transport-related operons similar to the *opp* operon indicates other systems for nutrient aquisition may be present that have not been well characterized in *M. hyosynoviae*. OppA has also been characterized as an important virulence factor and has been shown to function as a cellular adhesin in *M. hominis* (Oakley et al. 1999). It has also been demonstrated to function as the primary ecto-ATPase on the surface of the cell and can induce apoptosis in cell culture (Oakley et al. 1999; Hopfe and Henrich 2008). There is a large body of evidence that indicates many *Mycoplasma* species induce apoptosis (Oakley et al. 1999; Zhang and Lo 2007) and the release of nutrients associated with apoptosis may serve as a primary means of obtaining metabolites for many *Mycoplasma* species *in vivo*.

All seven strains of *M. hyosynoviae* characterized in this study also carry copies of genes encoding ftsY and ffh which serve to form a signal recognition particle pathway involved in GTP hydrolysis. Strain NPL7 encodes two copies of ffh. Homologs of these molecules have also been identified in *M. mycoides*, *M. genitalium*, and *M. pneumonia* (Macao et al. 1997) and these proteins likely play a pivotal role in the transport of GTP into the cell from the host environment.

Additionally, all seven strains of *M. hyosynoviae* encode three ECF (energy-coupling factor) transporter genes that appear to be linked together in an operon as well as a folT ECF transporter that is encoded at a more distal location within the genome. The three component genes encoded in the operon comprised two ATP-binding proteins and a transmembrane protein as has been reported for other organisms (Slotboom 2013). In conjunction with the *folT* gene, this set of genes likely controls the import of folate into the cell. *Mycoplasma* species often require folate supplementation for optimal growth (Bigland and Warenycia 1978) and the presence of this set of genes is indicative of *M. hyosynoviae*'s dependence on its host for procurement of folate. In our study, no supplemental folate was added to the medium for growth; however, the strains likely were able to scavenge this molecule from the serum component of the media.

All seven strains presented in this study also possess a complement of genes involved in cation transport. Procurement of iron may be accomplished through the use of a siderophore-mediated iron transport protein similar to that encoded by *M. pulmonis*. Acquisition of iron may also be accomplished through the use of a ZIP family permease present in all seven strains that can directly transport Fe^2+^, Mn^2+^, and Zn^2+^ into the cell. Permeases belonging to this family are also present in the genomes of *M. cynos* and *M. crocodyli*. Additionally, all seven strains possess a gene encoding a P-type, ATP driven Mg^2+^ transporter that would transport Mg^2+^ ions into the cell at the expense of ATP. Homologs of this protein are also present in the genomes of *M. mycoides* and *M. putrefaciens*.

### Purine synthesis

All the NPL strains possess the genes necessary to produce ATP for incorporation into RNA using a purine nucleoside phosphorylase to convert adenosine to adenine and then to adenosine monophosphate using adenine phosphoribosyltransferase. AMP could then be converted to ADP using adenlyate kinase and to ATP using pyruvate kinase before incorporation into RNA using an RNA polymerase holoenzyme. A similar mechanism can be used by these strains to produce GMP starting from guanosine with the added ability to use hypoxanthine-guanine phosphoribosyltransferase. The strains could then utilize guanylate kinase to convert GMP to GDP and pyruvate kinase to convert GDP to GTP before incorporation into RNA using RNA polymerase.

*Mycoplasma hyosynoviae* appears to lack the ability to produce dGTP and dATP for incorporation into DNA from guanosine and adenosine, however, it lacks the enzymes 5′-ribonucleotide phosphohydrolase, deoxyguanosine kinase, and deoxyadenosine kinase. Thus, *M. hyosynoviae* appears to begin DNA synthesis by importing dGMP and dAMP before converting these to dNTP through the use of guanylate kinase and adenylate kinase as well as pyruvate kinase. This of course is followed by incorporation into DNA by DNA polymerase.

### Pyrimidine synthesis

Like most species of *Mycoplasma*, *M. hyosynoviae* strains lack a complete pathway for synthesis of uracil triphosphate for incorporation into RNA. Similarly, the strains studied here lack an identifiable means of producing dTTP and dCTP for incorporation into DNA. The lack of such a mechanism is a common feature of many species within the Mollicutes class. It has been suggested that Mollicutes produce dTTP and dCTP through the action of low-specificity kinases (Bizarro and Schuck 2007) such as has been reported for the adenylate kinase of *M. tuberculosis* (Meena et al. 2003). All seven strains do possess the gene for CTP synthase and therefore can convert UTP to CTP for incorporation into RNA. Interestingly, all seven of the strains included in this study possess a complete pathway to convert cytosine and uracil to UDP, however, they lack any pathway that can convert UDP to UTP. Additionally, all seven strains possess the gene for thymidylate kinase which converts dUMP to dUDP and all seven also possess the gene for cytidine deaminase which catalyzes the conversion of deoxycytidine to deoxyuridine, but there is no path forward in this pathway toward dTTP. Instead, the genes present appear to direct a large portion of the deoxycytidine and deoxyuridine that the organism harvests from the host toward the production of uracil through the action of the enzyme purine nucleoside phosphorylase. Also, all seven strains possess five different enzymes capable of converting pseudouridine 5′ phosphate to 5-ribosyl uracil (pseudouridine) including pseudouridine synthase A, B, C, and D as well as tRNA pseudouridine synthase. The reason for the emphasis on production of pseudouridine is likely due to a strict need for these residues to be mounted on rRNA in the region of the ribosome that exhibits peptidyl transferase activity. Many of these enzymes have been shown to perform homologous functions in *E. coli*. In terms of pseudouridine synthesis they have been shown to differ in specificity for different substrates and function to add pseudouridine at different locations in rRNA (Conrad et al. 1999). *Mycoplasma hyosynoviae* is an accomplished parasite that efficiently harvests amino acids from its environment. It would need a robust system for manufacturing rRNA and complete ribosomes to convert amino acids harvested from the host environment into polypeptides. The pseudouridine harvested from this pathway is likely used in the manufacture of those ribosomes.

### Heat-shock response and other stress response proteins

All seven strains included in this study appear to possess a number of genes thought to respond to stress induced by heat shock. These include three genes of the DnaK cluster- *dnaK*, *grpE*, and *hrcA*. This cluster of proteins is known to aid in resolving protein aggregates and refolding proteins during heat shock in *M. genitalium* (Musatovova et al. 2006), while HrcA is a negative regulator that represses transcription of these genes. The strains also possess a number of other genes that encode proteins associated with the heat-shock response including LepA, RsmE, DnaJ, SmpB, and RsmI. LepA is a translation elongation factor responsible for back-translocating ribosomes after an incorrect translation that is universally present in all prokaryotes (Qin et al. 2006). In combination with GrpE, the DnaJ protein acts as a regulatory chaperone that regulates the binding of DnaK to newly transcribed proteins (Musatovova et al. 2006). With the exception of strain NPL7, all of the strains possess only one copy of these genes, whereas NPL7 encodes two copies of both RsmE and SmpB. RsmE is a ribosomal RNA methyltransferase that methylates the U1498 position of the 16S rRNA subunit in *E.coli* (Basturea et al. 2006). However, its role in the heat-shock response, particularly for *Mycoplasma* species is not well characterized. SmpB is an RNA-binding protein that binds to tmRNA to prevent degradation by RNAse R allowing the tmRNA to aid ribosomes that are stalled on an mRNA (Shin and Price 2007). Polypeptides that are stalled in this way are usually tagged for degradation. As ribosomal stalling occurs more frequently during heat shock, the SmpB protein plays a pivotal role in relieving stress from temperature extremes. The fact that strain seven encodes two copies of these genes is likely a result of gene duplication and it may give this strain some advantage during heat stress. The strains also encode two proteins related to a response to oxidative stress, YggF and RsmE, and strain seven encodes two copies of RsmE. Both of these proteins are known to aid in resolving damage to nucleic acids by silencing RNA transcripts and damaged tRNAs during oxidative stress. In light of the capacity for this organism to survive during an ongoing immune response these genes could prove important in limiting damage to the pathogen caused by secretion of hydroxyl radicals by infiltrating neutrophils at the site of infection.

### Antibiotic resistance and putative virulence factors

All seven strains included in this study encode the topoisomerase genes *parC* and *parE* as well as the DNA gyrase genes *gyrA* and *gyrB*. Specific mutations in these molecules have been implicated as the cause of fluoroquinolone resistance in *M. hominis* (Bebear et al. 1998). Several differences are present in the *parC* gene between the nonresistant *M. hominis* strain and the *M. hyosynoviae* strains reported here. Many of these are located in the region of the *parC* gene that has been associated with resistance in fluoroquinolone-resistant strains of *M. hominis* (Bebear et al. 1998). This is an interesting finding as enrofloxacin is often used to treat swine for respiratory symptoms. Further testing will be required to determine if these strains are actually resistant to fluoroquinolone antibiotics. At the very least, our sequence analysis indicates that *M. hyosynoviae* possesses the molecular machinery necessary to gain resistance to antibiotics in the fluoroquinolone class including those that target topoisomerase IV such as ciprofloxacin and that changes may be present in the strains discussed here that could impact resistance.

All seven strains encode elongation factor Tu which has been shown to be expressed on the surface of *M. pneumoniae* and *M. genitalium* and is known to play an important role in tissue-specific adhesion to host cells during infection with these species (Balasubramanian et al. 2009). All seven strains also possess a copy of elongation factor G which has been shown to be secreted and immunogenic during infection with *M. mycoides* in cattle (Jores et al. 2009). However, its role in pathogenesis currently remains unknown. In the strains presented here, elongation factor G is encoded in an operon alongside the genes for small subunit ribosomal proteins S12p and S7p. Mutations in the S12p gene have been linked to streptomycin resistance in *M. smegmatis* (Kenney and Churchward 1994); however, whether any of the strains involved in this study is streptomycin resistant remains unknown at this time.

All seven of the strains presented in this study encode a complete *infC*-*rpmI*-*rplT* operon consisting of translation initiation factor 3 (*infC*), and two large subunit ribosomal proteins L35p (*rpmI*) and *rplT*. NPL5 encodes two copies of this operon within its genome. This operon has been reported to function as an alternative system for restoration of functions associated with the sensor kinase *lemA* in *Pseudomonas syringae* (Kitten and Willis 1996). *lemA* autophosphorylation leads to expression and secretion of proteases into the extracellular environment. All seven of our strains also encode a *lemA* gene and a homologous system could be functioning in *M. hyosynoviae*. Certainly, it would make sense for a parasite such as *M. hyosynoviae* to encode a system that could maintain the secretion of proteases to the host environment in the absence of *lemA* activation so that digested proteins could be obtained at will and used in metabolism.

Also of interest is the presence of a gene encoding a virulence-associated protein D (*vapD*) homolog within the genome of these isolates. This virulence factor is distributed throughout many different species of bacteria and it has been assigned a number of different roles in virulence mechanisms including roles as a toxin, a plasmid maintenance protein, an endoribonuclease, and a role in acid metabolism (Kwon et al. 2012). Its role in the pathogenesis of species within the Mollicutes class has not been described previously; however, the gene encoding this protein is present in several species of *Mycoplasma* including *M. arthritidis*, *M. orale*, and *M. hominis*.

In addition to these putative virulence factors, every strain of *M. hyosynoviae* sequenced in the present study also possesses a copy of a protein that exhibits some homology to the MAA1 protein of *M. arthritidis* and a hypothetical protein from *M. orale* (Data S1). In *M. arthritidis*, MAA1 has been shown to mediate adhesion of bacterial cells to synovial membranes. As mentioned previously, *M. hyosynoviae* also encodes a number of other proteins that could serve in attachment of the bacteria to host cells and tissues including *oppA* and these could be used by the bacterium either for attachment within the tonsils or for adhesion in joint tissue.

### Posttranslational modifications

*Mycoplasma hyosynoviae* appears to possess two genes encoding proteins with known abilities to posttranslationally modify proteins by adding a lipid moiety, *lgt* and *lspA*. NPL7 encodes two copies of *lgt*. Interestingly, none of the strains reported in our study possess an apolipoprotein *N*-acetyltransferase (*Lnt*) gene which might indicate that none of the lipoproteins made by *M. hyosynoviae* would be *N*-acylated, rather the pathway would end with a cleaved lipoprotein with an amine group on the target cysteine. In *E. coli*, deletion of the *Lnt* gene results in lipoprotein accumulation within the cell as the *N*-acylation signal is required for transport of lipoproteins to the cell membrane. This phenotype eventually results in death for the cell. The absence of *Lnt* in these strains may indicate that *M. hyosynoviae* possesses another protein with a homologous function that we were unable to identify in our annotation or it may indicate that *M. hyosynoviae* is able to transport lipoproteins to the cell membrane in the absence of an *N*-acylation signal. A recent study in the Mollicutes species *Acholeplasma laidlawii* has demonstrated that lipoproteins derived from this species were *N*-acylated despite the absence of an *lnt* homolog (Serebryakova et al. 2011). Two other studies concerning lipoproteins derived from several other species of *Mycoplasma* demonstrated that these strains lacked an *N*-acyl modification (Muhlradt et al. 1998; Jan et al. 2001). Thus, a determination as to whether *M. hyosynoviae* is capable of producing *N*-acylated lipoproteins will need to await further analysis.

All seven strains encode a copy of *msrA*, a peptide methionine sulfoxide reductase that reduces methionine sulfoxide residues to methionine. In *M. genitalium* this protein has been shown to increase the virulence of wild-type strains and it is thought that this is caused by an increased ability of the bacterium to protect itself from oxidative damage from peroxide radicals produced during the immune response (Dhandayuthapani et al. 2001).

### Clustered regularly interspersed palindromic repeats

All of the strains contain potential clustered regularly interspersed palindromic repeat (CRISPR) elements. However, only strains NPL1 and NPL2, which encode a CRISPR-associated Cas system with *cas1*, *cas2*, and *csn1* in a continuous operon, contain CRISPRs with a large number of repeats. This structure is consistent with a Nmeni subtype CRISPR system (Haft et al. 2005). Strains NPL3, NPL4, and NPL5 contain a remnant of a CRISPR element with only five repeat sequences. As mentioned previously, these elements are located within the coding region for a *F. magna* hypothetical protein homolog encoded within the prophage genome identified in these three strains. All seven strains also possess conserved elements with only one set of repeats that were identified as possible CRISPRs by the CRISPR finder utility. These are likely either remnants of ancestral CRISPR elements or were simply misidentified by the program as they are conserved between all seven strains, located within the same regions of the genome, and with the exception of elements in strains 2 and 6 which have been partially incorporated into neighboring genes appear to be encoded outside of existing ORFs. A BLAST search using the spacer sequences from the confirmed CRISPR sequences in strains 1 and 2 revealed little homology to any phage or transposable element in either the NCBI or CRISPRfinder database implying that the types of phage and transposable elements this system protects against have yet to be discovered.

**Figure 4:**
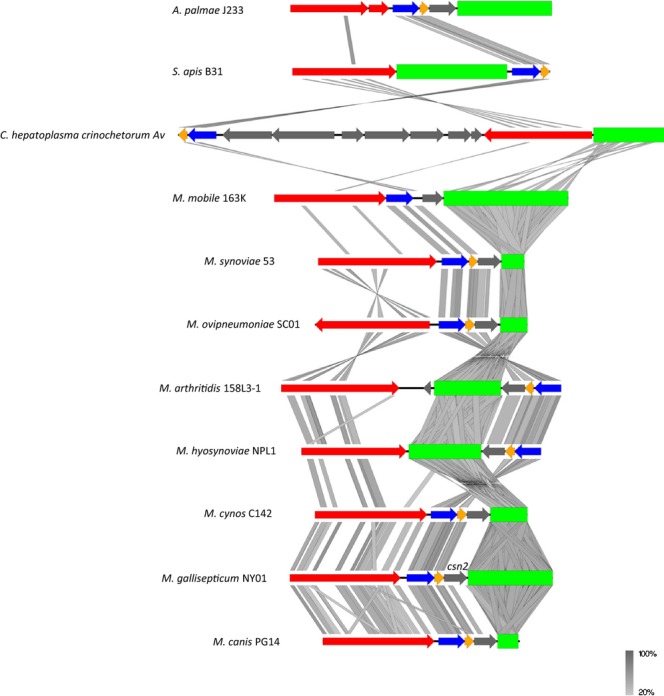
Comparison of CRISPR (clustered regularly interspaced short palindromic repeats) loci from Mollicutes species. Complete CRISPR loci from Mollicutes species were compared using Easy Fig 2.1. Coding sequences marked in red correspond to *csn1* or *cas9* genes. *cas2* sequences are colored blue. *cas1* sequences are displayed in yellow. CRISPR arrays are green in color. ORFs (open reading frames) marked in gray are either genes with functions unrelated to the functional CRISPR/Cas system or encode hypothetical proteins. Gray shading is used to indicate homology between sequences based on a TBlastX comparison.

When we compared the CRISPR locus of the NPL1 strain to loci derived from several other Mollicutes species (Fig.4), the structure of the operon bore the greatest similarity to *M. arthritidis* due to the orientation and position of the *cas* genes surrounding the array. In most Mollicutes species the *cas1* and *cas2* genes appear to be encoded sequentially following *csn1* or *cas9* and the CRISPR array is positioned downstream of *cas2*. However, in *M. arthritidis* and *M. hyosynoviae cas1* and *cas2* are encoded in the opposite direction as *csn1* and are located on the opposite side of the CRISPR array. Interestingly, all of the *Mycoplasma* species included in our comparison encode a hypothetical protein immediately prior to the CRISPR array following *cas2*. The function of this protein has not yet been determined but it is likely to have some function in CRISPR biology and should be investigated further. Indeed, within *M. synoviae*, *M. ovipneumoniae*, *M. arthritidis*, and *M. hyosynoviae* portions of this gene are somewhat conserved indicating that conserved domains may be present in the encoded protein. The location of the gene is consistent with the placement of *csn2* within *M. gallisepticum* as described in a previous study (Leclerq et al. 2014); however, as homology in this gene is limited we were unable to assign a name to it. In contrast, *cas1* and *cas2* proteins appear to be more highly conserved across Mollicutes species, while *csn1* or *cas9* exhibits intermediate homology. Within the CRISPR arrays themselves some homology appears to be present, however, it is dispersed along the array and is primarily to be found within the repeat sequences at a low level of identity. Overall, we conclude that CRISPR loci within the Mollicutes species that we compared exhibit some important variation in overall architecture. The efficiency of each gene arrangement in terms of the CRISPR system's ability to protect its host from invasion by either new or previously encountered phage has yet to be tested in *Mycoplasma* species and may be of interest for future research.

In summary, the draft genome sequences of the seven NPL strains reported here paint a picture of *M. hyosynoviae* as an opportunistic parasite dependent on its host for numerous nutritional requirements. This is in concordance with other members of the *Mycoplasma* genus as most *Mycoplasma* species are heavily dependent on their hosts for fixation of nutrients. However, *M. hyosynoviae* possesses several unique adaptations that confer a selective advantage for growth in its environmental niche including a large arsenal of ribonucleases, several DNA repair mechanisms, and an enhanced ability to shield itself from damage due to oxidative and heat stressors. Importantly, these strains also possess the molecular machinery necessary to develop resistance against fluoroquinolone antibiotics and several nucleotide changes are present in the fluoroquinolone resistance determining region of their *parC* genes when compared against susceptible *M. hominis* strains. The strains presented here also encode several putative adhesins that have been implicated in attachment to host surfaces in several other *Mycoplasma* species.

Phage appear to constitute a major threat to *M. hyosynoviae* as several strains of phage were identified in this report including one consistently identified among three of the seven *M. hyosynoviae* strains reported here. Strain NPL4 appears to be one of the most heavily infected strains of *Mycoplasma* ever reported as it contains two complete as well as one incomplete prophage sequence within its genome. Nonetheless, *M. hyosynoviae* is not defenseless against invasion by phage. Two of the strains reported here contain a CRISPR-Cas system that should help confer resistance to infection upon these strains and indeed no prophage sequences were identified in the draft genomes of either of these strains. These two strains also possess a type III restriction and modification system that could increase the ability of these strains to defend themselves against invasion by phage. Additionally, all seven strains possess a type I restriction modification system that could be used for the same purpose.

The draft genomes and comparative analysis reported here should be of value to further research on this emerging swine pathogen. Further studies concerning the organism's ability to resist host defence and colonize joint tissue should be undertaken to enable the design of interventions that could prevent disease caused by *M. hyosynoviae*. These efforts may prove to be particularly important in light of the potential for this organism to develop resistance against fluoroquinolone antibiotics.

## Supporting Information

**Table S1.** Table of locus tags for genes discussed.

Data S1 Alignment of MAA1 sequences from *Mycoplasma hyosynoviae* strains to the MAA1 sequence of *M. arthritidis* and a hypothetical protein sequence from *M. orale*. An alignment of MAA1 sequences generated using ClustalW2 and visualized using Boxshade. Reference sequences were obtained from the NCBI database with the following accession numbers: *M. arthritidis* MAA1 (NCBI: AAF03943.1); *M. orale* hypothetical protein (NCBI: WP_022936243.1). Accession numbers for putative MAA1 sequences derived from *M. hyosynoviae* are listed in Table S1.

**Figure S1.** Contig alignment in Mauve. Contig boundaries are marked with black lines.
