# Supplementary material for: Comparative genomic analysis of seven Mycoplasma hyosynoviae strains
Source: Microbiologyopen. 2015 Feb 18;4(2):343–59. doi: 10.1002/mbo3.242 (PMC4398514; doi:10.1002/mbo3.242)
Supplement: Data S1 — Alignment of MAA1 sequences from Mycoplasma hyosynoviae strains to the MAA1 sequence of M. arthritidis and a hypothetical protein sequence from M. orale. An alignment of MAA1 sequences generated using ClustalW2 and visualized using Boxshade. Reference sequences were obtained from the NCBI database with the following accession numbers: M. arthritidis MAA1 (NCBI: AAF03943.1); M. orale hypothetical protein (NCBI: WP_022936243.1). Accession numbers for putative MAA1 sequences derived from M. hyosynoviae are listed in Table S1. [file mbo30004-0343-sd2.docx]

*M. arthrit.* 1 MKKFKKNLSLLTVSILGTTLFATSVVAAACD-------------------
*M. orale* 1 ---MKKNILLLSTSLL--VLPSMPLILQSCENKETKEMSNKLNE---EIK
*M. hy.* NPL1 1 MIK-KKNLLIISIPLLTSTPLA---FSVACKDTKAEQLQKAISAYQTELK
*M. hy.* NPL2 1 MIK-KKNLLIISIPLLTSTPLA---FSVACKDTKAEQLQKAISAYQTELK
*M. hy.* NPL3 1 MIK-KKNLLIISIPLLTSTPLA---FSVACKDAKAEQLQKAISAYQTELK
*M. hy.* NPL4 1 MIK-KKNLLIISIPLLTSTPLA---FSVACKDAKAEQLQKAISAYQTELK
*M. hy.* NPL5 1 MIK-KKNLLIISIPLLTSTPLA---FSVACKDAKAEQLQKAISAYQTELK
*M. hy.* NPL6 1 MIK-KKNLLIISIPLLTSTPLA---FSVACKDAKAEQLQKAISAYQTELK
M. hy. NPL7 1 MIK-KKNLLIISIPLLTSTPLA---FSVACKDAKAEQLQKAISAYQTELK
 ... ***........*...... ....*....................

*M. arthrit.* 32 -----------------------DTKKKPEEPKKE-DSKQKPDQKQEQGQ
*M. orale* 43 VANSLIDQIGSLPKYETLQSDVTELKKLIEESKKVLSSKKSANELKVQFE
*M. hy.* NPL1 47 VAEQLIKELEDATKYPQLQNDLTMLKKEVEAIKKEFEKAKTSTTELQKLI
*M. hy.* NPL2 47 VAEQLIKELEDATKYPQLQNDLTMLKKEVEAIKKEFEKAKTSTTELQKLI
*M. hy.* NPL3 47 VAEQLIQELEDATKYPQLQNDLTMLKKEVEAIKKEFEKAKTSTIELQKLI
*M. hy.* NPL4 47 VAEQLIQELEDATKYPQLQNDLTMLKKEVEAIKKEFEKAKTSTIELQKLI
*M. hy.* NPL5 47 VAEQLIQELEDATKYPQLQNDLTMLKKEVEAIKKEFEKAKTSTIELQKLI
*M. hy.* NPL6 47 VAEQLIQELEDATKYPQLQNDLTMLKKEVEAIKKEFEKAKTSTIELQKLI
*M. hy.* NPL7 47 VAEQLIQELEDATKYPQLQNDLTMLKKEVEAIKKEFEKAKTSTIELQKLI
 .........................**..*..**................

*M. arthrit.* 58 EI-------------------TELNNWLKSMSLKIVDGKIDAFYKAIEAK
*M. orale* 93 KLVKM-VGSVYNKKLQIDNRDTTFDNLDASNSLSIISDKLDQFYKAIADK
*M. hy.* NPL1 97 TKIKIKTQTTQQKKHEEDSRDKTFDSIDASNSLEIISENASDFYESIRQG
*M. hy.* NPL2 97 TKIKIKTQTTQQKKHEEDSRDKTFDSIDASNSLEIISENASDFYESIRQG
*M. hy.* NPL3 97 TKIKLKTQATQQKKHEEDSRDKTFDSIDASNSLEIISENASDFYEAIRQG
*M. hy.* NPL4 97 TKIKLKTQATQQKKHEEDSRDKTFDSIDASNSLEIISENASDFYEAIRQG
*M. hy.* NPL5 97 TKIKLKTQATQQKKHEEDSRDKTFDSIDASNSLEIISENASDFYEAIRQG
*M. hy.* NPL6 97 TKIKLKTQATQQKKHEEDSRDKTFDSIDASNSLEIISENASDFYEAIRQG
*M. hy.* NPL7 97 TKIKLKTQATQQKKHEEDSRDKTFDSIDASNSLEIISENASDFYEAIRQG
 .............................*.**.*.......**..*...

*M. arthrit.* 89 KDFYYSYSSKKLIAVEKGKRPNWREENEELLSLEGEFASNKHQIVNDEKP
*M. orale* 142 KDFYYDYKNNKMIAVEAGKRPNWQETNEYLIDVKIKDLANDLQIANAKEP
*M. hy.*NPL1 147 NDFYFDRKAYKLVAVEKGKRPNWTEVQKYLVNVNIKDIASDLQLANHSEP
*M. hy.*NPL2 147 NDFYFDRKAYKLVAVEKGKRPNWTEVQKYLVNVNIKDIASDLQLANHTEP
*M. hy.*NPL3 147 NDFYFDRKAYKLVAVEKGKRPNWTEVQKYLVNVNIKDIASDLQLANHTEP
*M. hy.*NPL4 147 NDFYFDRKAYKLVAVEKGKRPNWTEVQKYLVNVNIKDIASDLQLANHTEP
*M. hy.*NPL5 147 NDFYFDRKAYKLVAVEKGKRPNWTEVQKYLVNVNIKDIASDLQLANHTEP
*M. hy.*NPL6 147 NDFYFDRKAYKLVAVEKGKRPNWTEVQKYLVNVNIKDIASDLQLANHTEP
*M. hy.*NPL7 147 NDFYFDRKAYKLVAVEKGKRPNWTEVQKYLVNVNIKDIASDLQLANHTEP
 .***......*..***.******.*....*............*..*...*

*M. arthrit.*139 TYTDSKGMVKLSSLIRYEVK-DGKIIFYFKGAIHDHKGAHKISTEVHKME
*M. orale* 192 TY-ESHGKIQVSGKIDYEINANGEIIFSYKIGFY-NRGNPKISETVFTTN
*M. hy.*NPL1 197 LY----GGNKISGKIDYEIK-DKEIIFKYRVAFFN-GGNPKVSEKLYETN
*M. hy.*NPL2 197 LY----GGNKISGKIDYEIK-DKEIIFKYRAAFFN-GGNPKVSEKLYETN
*M. hy.*NPL3 197 LY----GGNKISGKIDYEIK-DKEIIFKYRAAFFN-GGNPKVSEKLYETN
*M. hy.*NPL4 197 LY----GGNKISGKIDYEIK-DKEIIFKYRAAFFN-GGNPKVSEKLYETN
*M. hy.*NPL5 197 LY----GGNKISGKIDYEIK-DKEIIFKYRAAFFN-GGNPKVSEKLYETN
*M. hy.*NPL6 197 LY----GGNKISGKIDYEIK-DKEIIFKYRAAFFN-GGNPKVSEKLYETN
*M. hy.*NPL7 197 LY----GGNKISGKIDYEIK-DKEIIFKYRAAFFN-GGNPKVSEKLYETN
 .* *....*..*.**.. ...***........ .*..*.*.......
*M. arthrit.*188 FPR------QSLASNFEEYVKNLQFSYP---NLEETLLDDVDIDKVHPSE
*M. orale* 240 LGKPLSEEEQKEIAKLEEAEKNTTFSYSG--DISNTLLKDADLSKV--VA
*M. hy.*NPL1 241 LGEILSQEQLEKLAKLEEAESKTTFEYKGPKQLEETYLSEVKDEEI--LS
*M. hy.*NPL2 241 LGEILSQEQLEKLAKLEEAESKTTFEYKGPKQLEETYLSEVKDEEI--SS
*M. hy.*NPL3 241 LGEILSQEQLEKLAKLEEAESKTTFEYKGPKQLEETYLSEVKDEEI--SS
*M. hy.*NPL4 241 LGEILSQEQLEKLAKLEEAESKTTFEYKGPKQLEETYLSEVKDEEI--SS
*M. hy.*NPL5 241 LGEILSQEQLEKLAKLEEAESKTTFEYKGPKQLEETYLSEVKDEEI--SS
*M. hy.*NPL6 241 LGEILSQEQLEKLAKLEEAESKTTFEYKGPKQLEETYLSEVKDEEI--SS
*M. hy.*NPL7 241 LGEILSQEQLEKLAKLEEAESKTTFEYKGPKQLEETYLSEVKDEEI--SS
 ................**......*.*........*.*........ ..

*M. arthrit.*229 DLKEGYEFAKKTIVKEPETN--TLSILYSIRKKDIDYKSK-NYVFNLKGW
*M. orale* 286 NVPEGFEIESKKMVPNDEAGFYELTILFKLKSKKVNVSSKKNKEFALKGW
*M. hy.*NPL1 289 KVPEGFEIAKQKIVKNSDVGFYELTILFKLKIKGTDIVSKKNKQFIIIGW
*M. hy.*NPL2 289 KVPEGFEIAKQKIVKNSDVGFYELTILFKLKIKGTDIVSKKNKQFIIIGW
*M. hy.*NPL3 289 KVPEGFEIAKQKIVKNSDVGFYELTILFKLKIKGTDIVSKKNKQFIIIGW
*M. hy.*NPL4 289 KVPEGFEIAKQKIVKNSDVGFYELTILFKLKIKGTDIVSKKNKQFIIIGW
*M. hy.*NPL5 289 KVPEGFEIAKQKIVKNSDVGFYELTILFKLKIKGTDIVSKKNKQFIIIGW
*M. hy.*NPL6 289 KVPEGFEIAKQKIVKNSDVGFYELTILFKLKIKGTDIVSKKNKQFIIIGW
*M. hy.*NPL7 289 KVPEGFEIAKQKIVKNSDVGFYELTILFKLKIKGTDIVSKKNKQFIIIGW
 ...**.*......*.........*.**.....*.....**.*..*...**

*M. arthrit.*276 KKSSEMLKKIEEAKNKINEEINKLDIKILNEKAYQNIKETNSHLNFEGKS
*M. orale* 336 KKTPEKIAEEEQAKKIIEEETKNIKAYISNETAYQDIINRNAIAQENHTP
*M. hy.*NPL1 339 KKTPEKIAEEEKAKKEIEEQTKTIKVYVSNEKAYQDIIVRQKPTQENAQP
*M. hy.*NPL2 339 KKTPEKIAEEEKAKKEIEEQTKTIKVYVSNEKAYQDIIVRQKPTQENAQP
*M. hy.*NPL3 339 KKTPEKIAEEEKAKKEIEEQTKTIKVYVSNEKAYQDIIVRQKPTQENAQP
*M. hy.*NPL4 339 KKTPEKIAEEEKAKKEIEEQTKTIKVYVSNEKAYQDIIVRQKPTQENAQP
*M. hy.*NPL5 339 KKTPEKIAEEEKAKKEIEEQTKTIKVYVSNEKAYQDIIVRQKPTQENAQP
*M. hy.*NPL6 339 KKTPEKIAEEEKAKKEIEEQTKTIKVYVSNEKAYQDIIVRQKPTQENAQP
*M. hy.*NPL7 339 KKTPEKIAEEEKAKKEIEEQTKTIKVYVSNEKAYQDIIVRQKPTQENAQP
 **..*.....*.**..*.*..........**.***.*.............

*M. arthrit.*326 NFATGSYDVNLFKLEYVSVKFNETS--KKIEVDVKLSIILDDSISVSKKL
*M. orale* 386 NFVISGYDSSKFIATATEVKVEEVAGKKKITISYEIHAIVNKDIKVLKTN
*M. hy.*NPL1 389 NFVLNGY-SNLFHATVVEVKVEDEGGKKKITVTYEIWAKANQSIKIKKQN
*M. hy.*NPL2 389 NFVLNGY-SNLFHATVVEVKVEDEGGKKKITVTYEIWAKANQSIKIKKQN
*M. hy.*NPL3 389 NFVLNGY-SNLFHATVVEVKVEDEGGKKKITVTYEIWAKANQSIKIKKQN
*M. hy.*NPL4 389 NFVLNGY-SNLFHATVVEVKVEDEGGKKKITVTYEIWAKANQSIKIKKQN
*M. hy.*NPL5 389 NFVLNGY-SNLFHATVVEVKVEDEGGKKKITVTYEIWAKANQSIKIKKQN
*M. hy.*NPL6 389 NFVLNGY-SNLFHATVVEVKVEDEGGKKKITVTYEIWAKANQSIKIKKQN
*M. hy.*NPL7 389 NFVLNGY-SNLFHATVVEVKVEDEGGKKKITVTYEIWAKANQSIKIKKQN
 **....* ...*......**.......***.............*...*..

*M. arthrit.*374 I-IEGNYANGNINPHNLTEEDQKKYLSDELEKMTVHPYYSKDKTYIERLN
*M. orale* 436 IQVETNYNKDVINPHNLSAEECKNFLTDAIKDEKICPFYSKDKTYIEKLN
*M. hy.*NPL1 438 VNVETNYNNDTTNPHNLTEEEQKKYLEDAIKDVKIIPFYSKDKTYIEKLR
*M. hy.*NPL2 438 VNVETNYNNDTTNPHNLTEEEQKKYLEDAIKDVKIIPFYSKDKTYIEKLR
*M. hy.*NPL3 438 VNVETNYNNDTTNPHNLTEEEQKKYLEDAIKDVKIIPFYSKDKTYIEKLR
*M. hy.*NPL4 438 VNVETNYNNDTTNPHNLTEEEQKKYLEDAIKDVKIIPFYSKDKTYIEKLR
*M. hy.*NPL5 438 VNVETNYNNDTTNPHNLTEEEQKKYLEDAIKDVKIIPFYSKDKTYIEKLR
*M. hy.*NPL6 438 VNVETNYNNDTTNPHNLTEEEQKKYLEDAIKDVKIIPFYSKDKTYIEKLR
*M. hy.*NPL7 438 VNVETNYNNDTTNPHNLTEEEQKKYLEDAIKDVKIIPFYSKDKTYIEKLR
 ...*.**.....*****..*..*..*.*........*.*********.*.
*M. arthrit.*423 DNELSDRSFWINNKN-KSLTYTFGKVEKTLEGKYNVEVTTSFFDWNESPR
*M. orale* 486 NLNLSNKSFFIEGKKLNNLIYNFGNVYKK-DNKYYVEAELSFAYWPGSPK
*M. hy.*NPL1 488 NEHLTNKSFFIQGKKYQNLEYQYGNVVKNGE-KFEVEVTMSFSYWSQSPK
*M. hy.*NPL2 488 NEHLTNKSFFIQGKKYQNLEYQYGNVVKNGE-KFEVEVTMSFSYWSQSPK
*M. hy.*NPL3 488 NEHLTNKSFFIQGKKYQNLEYQYGNVVKNGE-KFEVEVTMSFSYWSQSPK
*M. hy.*NPL4 488 NEHLTNKSFFIQGKKYQNLEYQYGNVVKNGE-KFEVEVTMSFSYWSQSPK
*M. hy.*NPL5 488 NEHLTNKSFFIQGKKYQNLEYQYGNVVKNGE-KFEVEVTMSFSYWSQSPK
*M. hy.*NPL6 488 NEHLTNKSFFIQGKKYQNLEYQYGNVVKNGE-KFEVEVTMSFSYWSQSPK
*M. hy.*NPL7 488 NEHLTNKSFFIQGKKYQNLEYQYGNVVKNGE-KFEVEVTMSFSYWSQSPK
 ...*...**.*..*....*.*..*.*.*... *..**...**..*..**.

*M. arthrit.*472 ETKKVLIDLEKLGIDIHNKIREQNNQAPLEDKKAPSGTVEENIDPDLNTE
*M. orale* 535 VKIEKEIDLEKLGVNILNEGKNESNK--IQDIEAPTGTITEQFEPTLDLS
*M. hy.*NPL1 537 VKVKKEIDLSILGVDEVNKSKPDGNK--IQDIEAPSATINDFFEPTLDIN
*M. hy.*NPL2 537 VKVKKEIDLSILGVDEVNKSKPDGNK--IQDIEAPSATINDFFEPTLDIN
*M. hy.*NPL3 537 VKVKKEIDLSILGVDEVNKSKPDGNK--IQDIEAPSATINDFFEPTLDIN
*M. hy.*NPL4 537 VKVKKEIDLSILGVDEVNKSKPDGNK--IQDIEAPSATINDFFEPTLDIN
*M. hy.*NPL5 537 VKVKKEIDLSILGVDEVNKSKPDGNK--IQDIEAPSATINDFFEPTLDIN
*M. hy.*NPL6 537 VKVKKEIDLSILGVDEVNKSKPDGNK--IQDIEAPSATINDFFEPTLDIN
*M. hy.*NPL7 537 VKVKKEIDLSILGVDEVNKSKPDGNK--IQDIEAPSATINDFFEPTLDIN
 ......***..**....*......*. ..*..**..*......*.*...

*M. arthrit.*522 GFKETPSDDHNGGASSIIHLRNFIEDIKKQKLMLWDNEMLTQIKEKKY-T
*M. orale* 583 KFTDSDSDDANGGPAINPIKKNTLSYI--NSLVCLNKDLYNQMVSGNFGS
*M. hy.*NPL1 585 KFKDSPEDEYSGNGAIKVIKTNTLDKMK--DLVCWSKALYDQMASGDFGE
*M. hy.*NPL2 585 KFKDSPEDEYSGNGAIKVIKTNTLDKMK--DLVCWSKALYDQMASGDFGE
*M. hy.*NPL3 585 KFKDSPEDEYSGNGAIKVIKTNTLDKMK--DLVCWSKALYDQMASGDFGE
*M. hy.*NPL4 585 KFKDSPEDEYSGNGAIKVIKTNTLDKMK--DLVCWSKALYDQMASGDFGE
*M. hy.*NPL5 585 KFKDSPEDEYSGNGAIKVIKTNTLDKMK--DLVCWSKALYDQMASGDFGE
*M. hy.*NPL6 585 KFKDSPEDEYSGNGAIKVIKTNTLDKMK--DLVCWSKALYDQMASGDFGE
*M. hy.*NPL7 585 KFKDSPEDEYSGNGAIKVIKTNTLDKMK--DLVCWSKALYDQMASGDFGE
 .*.....*...*.........*...... .*.........*........

*M. arthrit.*571 LPFAQFFSYDKEKLELKSKVQFFSKSL----KMVDGQTAFIISGP--KEA
*M. orale* 631 KKLLQHLKYDSTTFEAKSNVYFLPHNTFLPKEMYSEYYAYVFSSPIYDQE
*M. hy.*NPL1 633 KYLLQNLKYDKDTYEKKDNI-YFLQRKGSPKELLGNHYVFIFSKPKYDQN
*M. hy.*NPL2 633 KYLLQNLKYDKDTYEKKDNI-YFLQRKGSPKELLGNHYVFIFSKPKYDQN
*M. hy.*NPL3 633 KYLLQNLKYDKDTYEKKDNI-YFLQRKGSPKELLGNHYVFIFSKPKYDQN
*M. hy.*NPL4 633 KYLLQNLKYDKDTYEKKDNI-YFLQRKGSPKELLGNHYVFIFSKPKYDQN
*M. hy.*NPL5 633 KYLLQNLKYDKDTYEKKDNI-YFLQRKGSPKELLGNHYVFIFSKPKYDQN
*M. hy.*NPL6 633 KYLLQNLKYDKDTYEKKDNI-YFLQRKGSPKELLGNHYVFIFSKPKYDQN
*M. hy.*NPL7 633 KYLLQNLKYDKDTYEKKDNI-YFLQRKGSPKELLGNHYVFIFSKPKYDQN
 ....*...**....*.*... .....................*.*.....

*M. arthrit.*615 DGKLTIKLTLMTLQDFKAKNFNSENMISRRVEIDQTTFAKDYIT-KKFLL
*M. orale* 681 TKKLTIKISVVSTNDLNAGN-TKDQVASKRVEVVANEFLQCNIT-K-YLF
*M. hy.*NPL1 682 TKELTIKVSVVSVNDLNAGN-SVEQVASKRINVKKDIFLNENDTYKTILF
*M. hy.*NPL2 682 TKELTIKVSVVSVNDLNAGN-SVEQVASKRINVKKDIFLNENDTYKTILF
*M. hy.*NPL3 682 TKELTIKVSVVSVNDLNAGN-SVEQVASKRINVKKDIFLNENDTYKTILF
*M. hy.*NPL4 682 TKELTIKVSVVSVNDLNAGN-SVEQVASKRINVKKDIFLNENDTYKTILF
*M. hy.*NPL5 682 TKELTIKVSVVSVNDLNAGN-SVEQVASKRINVKKDIFLNENDTYKTILF
*M. hy.*NPL6 682 TKELTIKVSVVSVNDLNAGN-SVEQVASKRINVKKDIFLNENDTYKTILF
*M. hy.*NPL7 682 TKELTIKVSVVSVNDLNAGN-SVEQVASKRINVKKDIFLNENDTYKTILF
 ...****.......*..*.* ......*.*.......*.....*.*..*.
*M. arthrit.*664 ERYIRSLRLLFDCVGKEDMKPSEVEDSNMVILESHLPSGIK--LIEKSSH
*M. orale* 728 TKSVFDLRLKGSTF--DQSKPA--SEVTMEILKQKLEEHYVGYVIENVEV
*M. hy.*NPL1 731 SEDVY-AKSKFKA-DEWDKKNKSVDHYTVEVVTKMLQDKLTGYTIFDVKV
*M. hy.*NPL2 731 SEDVY-AKSKFKA-DEWDKKNKSVDHYTVEVVTKMLQDKLTGYTIFDVKV
*M. hy.*NPL3 731 SEDVY-AKSKFKA-DEWDKKNKSVDHYTVEVVTKMLQDKLTGYTIFDVKV
*M. hy.*NPL4 731 SEDVY-AKSKFKA-DEWDKKNKSVDHYTVEVVTKMLQDKLTGYTIFDVKV
*M. hy.*NPL5 731 SEDVY-AKSKFKA-DEWDKKNKSVDHYTVEVVTKMLQDKLTGYTIFDVKV
*M. hy.*NPL6 731 SEDVY-AKSKFKA-DEWDKKNKSVDHYTVEVVTKMLQDKLTGYTIFDVKV
*M. hy.*NPL7 731 SEDVY-AKSKFKA-DEWDKKNKSVDHYTVEVVTKMLQDKLTGYTIFDVKV
 ..... ....... .....*...............*........*.....

*M. arthrit.*712 D-MKDKKGTLTIKCKFSYNGVETGDMFYTIKGFKKQ
*M. orale* 774 DHSKSSEGKLLYKFSAKKDSFESFVLSLTISKFKK-
*M. hy.*NPL1 779 DHSEKDQGILKYEYTVKNDKFTSYPFKITVKNFKK-
*M. hy.*NPL2 779 DHSEKDQGILKYEYTVKNDKFTSYPFKITVKNFKK-
*M. hy.*NPL3 779 DHSEKDQGILKYEYTVKNDKFTSYPFKITVKNFKK-
*M. hy.*NPL4 779 DHSEKDQGILKYEYTVKNDKFTSYPFKITVKNFKK-
*M. hy.*NPL5 779 DHSEKDQGILKYEYTVKNDKFTSYPFKITVKNFKK-
*M. hy.*NPL6 779 DHSEKDQGILKYEYTVKNDKFTSYPFKITVKNFKK-
*M. hy.*NPL7 779 DHSEKDQGILKYEYTVKNDKFTSYPFKITVKNFKK-
 *......*.*..................*...***
