# Supplementary material for: Comparative genomic analysis of seven Mycoplasma hyosynoviae strains
Source: Microbiologyopen. 2015 Feb 18;4(2):343–59. doi: 10.1002/mbo3.242 (PMC4398514; doi:10.1002/mbo3.242)
Supplement: Supplementary file 3 — Figure S1. Contig alignment in Mauve. Contig boundaries are marked with black lines. [file mbo30004-0343-sd3.pptx]

## Slide 1
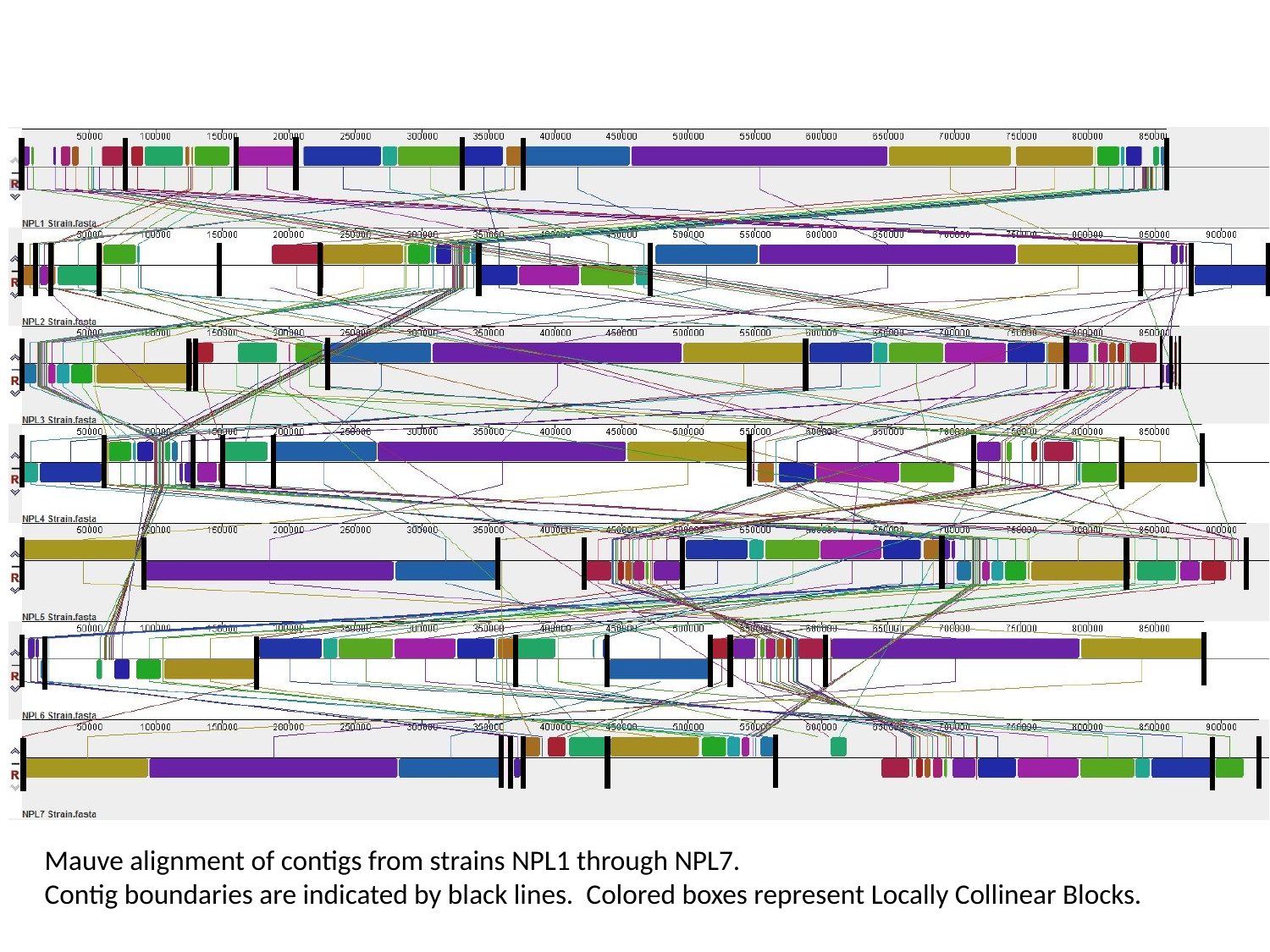

Mauve alignment of contigs from strains NPL1 through NPL7.
Contig boundaries are indicated by black lines. Colored boxes represent Locally Collinear Blocks.
